# Tumor secretome shapes the immune landscape during cancer progression

**DOI:** 10.1186/s13046-025-03302-0

**Published:** 2025-02-10

**Authors:** Jianqiang Yang, Sijia Tang, Nabil F. Saba, Chloe Shay, Yong Teng

**Affiliations:** 1 Department of Hematology and Medical Oncology, Emory University, 201 Dowman Dr, Atlanta, GA 30322 USA; 2 Winship Cancer Institute of Emory University, Atlanta, GA 30322 USA; 3 Wallace H. Coulter Department of Biomedical Engineering, Georgia Institute of Technology and Emory University, Atlanta, GA 30322 USA

**Keywords:** Tumor secretome, Tumor microenvironment, Tumor-immune cell interplay, Immunotherapy, Biomarkers

## Abstract

The focus of cancer immunotherapy has traditionally been on immune cells and tumor cells themselves, often overlooking the tumor secretome. This review provides a comprehensive overview of the intricate relationship between tumor cells and the immune response in cancer progression. It highlights the pivotal role of the tumor secretome - a diverse set of molecules secreted by tumor cells - in significantly influencing immune modulation, promoting immunosuppression, and facilitating tumor survival. In addition to elucidating these complex interactions, this review discusses current clinical trials targeting the tumor secretome and highlights their potential to advance personalized medicine strategies. These trials aim to overcome the challenges of the tumor microenvironment by designing therapies tailored to the secretome profiles of individual cancer patients. In addition, advances in proteomic techniques are highlighted as essential tools for unraveling the complexity of the tumor secretome, paving the way for improved cancer treatment outcomes.

## Introduction

The tumor secretome refers to the collection of molecules secreted by cells into the extracellular environment, including proteins, lipids, nucleic acids, metabolites, and other small molecules [1, 2]. Notably, the tumor secretome not only contributes to tumor growth and progression but also plays a pivotal role in shaping the tumor immune microenvironment [3]. Through tumor secretome-mediated interactions, tumor and immune cells can produce diverse effects, often promoting tumor survival and proliferation over an effective anti-tumor immune response [4]. Investigating the tumor secretome offers significant potential for deepening our understanding of cancer biology and developing novel therapeutic strategies. Recent advancements in this field have led to several noteworthy discoveries, highlighting the growing importance of the tumor secretome in cancer research.

## Tumor secretome and its functional components

**Fig. 1:**
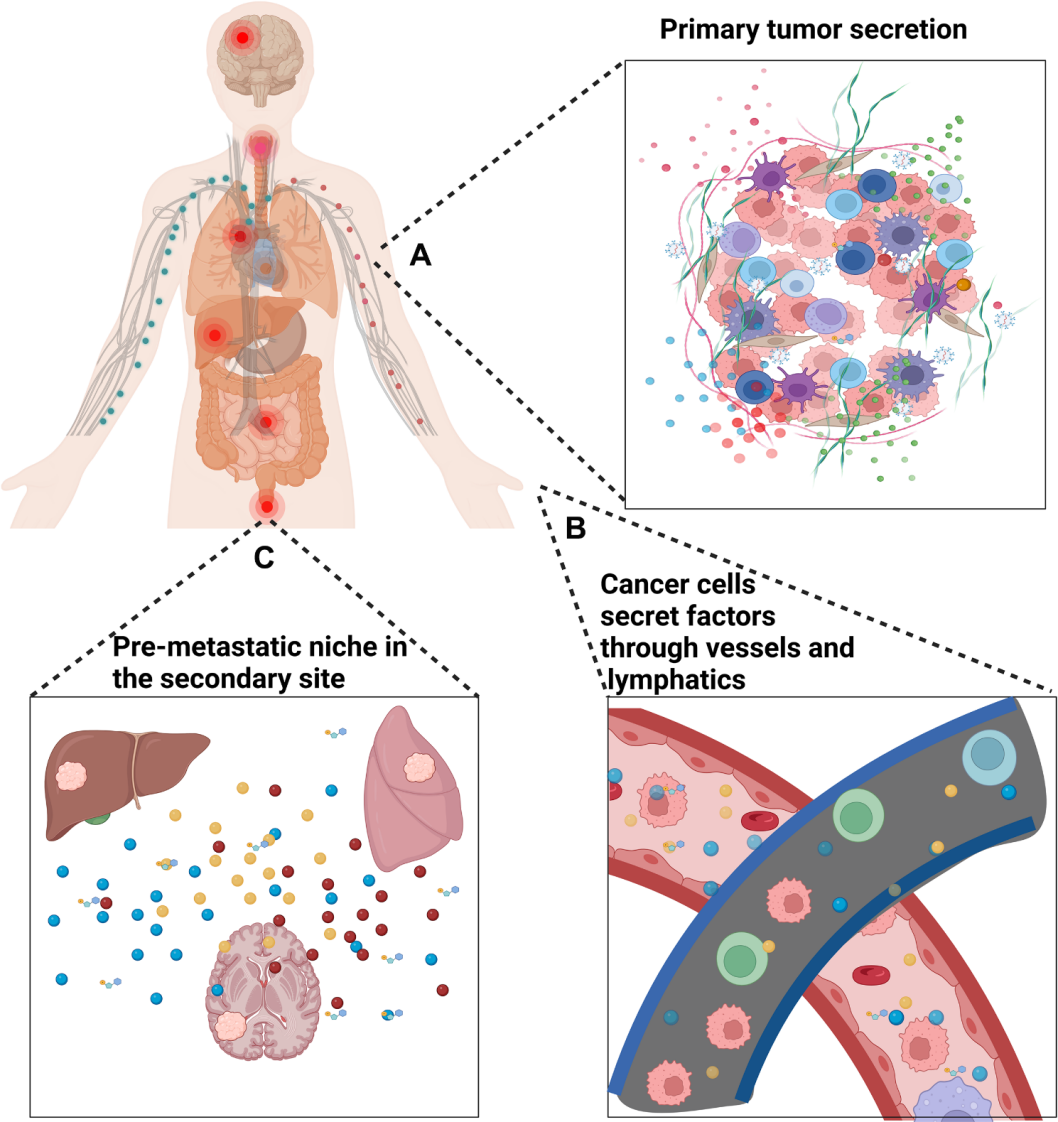
Impact of the tumor secretome on cancer development and progression. Components secreted by tumor cells play a critical role in the process of pre-metastasis and metastasis via the blood and lymphatic systems. In addition, such secreted substances often enter the blood or lymphatic system earlier than tumor cells to reach the secondary metastatic site

The tumor microenvironment (TME) is a complex milieu surrounding tumor cells, comprising a variety of cellular and non-cellular components that interact with tumor cells, significantly influencing tumor behavior and progression. Tumor cells communicate with surrounding cells through the secretion of biological substances, with approximately 13% of all human protein-coding genes dedicated to secreted proteins [5]. As tumors develop, these secreted proteins play a crucial role in regulating the behavior of other cells within the TME. Furthermore, under constant immune system surveillance, tumor cells have evolved mechanisms to suppress immune cell activity, enabling them to proliferate more effectively than normal cells. The substances secreted by tumor cells play a crucial role in modulating the immune response, influencing the function and activity of immune cells in ways that frequently support tumor development and progression (Fig. 1). Understanding these interactions is vital for developing strategies to counteract the immunosuppressive influences of the TME and improve cancer treatment outcomes.

### Cytokines

Cytokines are small signaling proteins essential for regulating immune responses. Tumor cells can secrete various cytokines that facilitate proliferation and enable immune evasion. Certain cytokines promote immunosuppression, fostering tumor growth and survival. Transforming growth factor beta (TGF-β) is a crucial regulator of immune cell development and function [6]and is recognized as a key cytokine that induces potent immunosuppressive effects [7]. Tumor cells frequently secrete TGF-β to inhibit the activation and proliferation of T cells and natural killer (NK) cells. Additionally, TGF-β promotes regulatory T cell differentiation, further reinforcing immunosuppression within the TME. Interestingly, studies have also shown that TGF-β can promote the differentiation of certain inflammatory T cell populations, such as Th17, Th9, and resident-memory T cells, which have been associated with improved tumor control in some models. This dual role of TGF-β highlights its complex contribution to tumor progression across different cancers. Beyond immunosuppression, TGF-β is also implicated in tumor-promoting processes such as angiogenesis, fibrosis, and epithelial-mesenchymal transition (EMT) [8]. Consequently, developing strategies to block TGF-β or its signaling pathways to restore anti-tumor immunity remains challenging. Interleukin-10 (IL-10) is another anti-inflammatory cytokine that suppresses the production of pro-inflammatory factors and inhibits the cytotoxic and cytokine-releasing functions of T cells, NK cells, macrophages, and dendritic cells, thereby diminishing anti-tumor immune responses. Typically, IL-10 serves as a critical anti-inflammatory mediator that prevents an overactive immune response to pathogens and microbiota. However, tumor cells often secrete excessive amounts of IL-10, thereby leveraging its function to dampen effective anti-tumor immunity.

Apparently, tumor-derived cytokines play a crucial role in recruiting immunosuppressive cells to the TME. IL-8 is a chemokine that interacts with C-X-C motif chemokine receptor 1 (CXCR1) and CXCR2, both of which are G-protein coupled receptors (GPCRs), present on granulocytes, monocytes, and endothelial cells. This interaction enhances angiogenesis, attracts immunosuppressive cells to the tumor site, and is associated with a poorer prognosis [9]. Additionally, IL-8 has been shown to recruit neutrophils and myeloid-derived suppressor cells (MDSCs), which can inhibit T cell activity [10]. CCL2 is instrumental in attracting tumor-associated macrophages (TAMs) that adopt a pro-tumorigenic phenotype, supporting cancer cell survival, enhancing tumor cell invasion, and promoting angiogenesis. CCL2 also exerts TAM-independent effects on tumor cells and the TME, such as recruiting other myeloid subsets and non-myeloid cells, which sustains an immunosuppressive environment, stimulates tumor cell growth and motility, and further promotes angiogenesis [11].

Recent research has underscored the importance of modulating the major histocompatibility complex (MHC) in cancer immune evasion. Numerous studies have reported that the loss or downregulation of MHC class I is a pivotal mechanism by which tumor cells evade the immune system. This downregulation prevents the presentation of tumor-associated antigens on the cell surface, thereby reducing the cytotoxic activity of CD8 + T cells and impairing the adaptive immune response [12]. Tumor cells may enhance the production of cytokines that downregulate MHC expression, diminishing their detection by T cells and enabling unchecked proliferation.

IL-6 has been shown to downregulate MHC class II expression on dendritic cells via the STAT3 pathway [13]. Similarly, IL-10 reduces the presence of MHC Class II αβ peptide complexes on the plasma membrane of monocytes by affecting their trafficking and recycling [14]. CCL5 and CXCL8 also play roles in recruiting monocytes, neutrophils, and other leukocytes, which can differentiate into TAMs and tumor-associated neutrophils (TANs), both of which can assume pro-tumorigenic roles [15].

### Metabolites

An increasing body of research is revealing the critical role that metabolites play in tumor immunity. Tumor cells undergo significant metabolic reprogramming to meet the demands of rapid proliferation and survival under often hostile conditions. This reprogramming is fundamentally different from the metabolism of normal cells. Consequently, tumor cells have altered metabolic profiles compared to normal cells and these metabolic changes lead to the production and release of various metabolites that can significantly affect immune cell function and contribute to immune evasion.

Tumor cells predominantly rely on glycolysis for energy production, even in the presence of oxygen, which results in increased production of lactate. Lactate contributes to the acidification of the TME and plays a crucial role in regulating the functions of macrophages and lymphocytes in the process of immune suppression [16]. Acidic conditions impair T cell receptor signaling and cytokine production, reducing their anti-tumor effectiveness [17, 18]. By contrast, lactate favors FOXP3 + regulatory T (Treg) cells, which can take up and consume lactate to sustain their immunosuppressive functions in an acidic environment [19]. Additionally, lactate potentiates the M2 polarization of alternatively activated macrophages, promoting angiogenesis and tumorigenesis [20]. A study investigating melanoma patients revealed that elevated expression of glycolysis-related genes was associated with poorer progression-free survival following anti- programmed cell death protein 1 (PD-1) therapy. Additionally, the study demonstrated that diclofenac plays a dual role by reducing lactate secretion from tumor cells and enhancing anti-PD-1-induced T cell activity in vitro. This effect is achieved through the significant inhibition of monocarboxylate transporters (MCTs) 1 and 4, which are essential for lactate efflux from tumor cells [21].

Emerging studies have revealed that altered amino acid metabolism is tightly linked to tumor outgrowth, metastasis, and therapeutic resistance by governing the fate of various immune cells [22]. Tumor cells regulate immunity through the regulation of amino acid metabolism. Tumor cells can upregulate amino acid-metabolizing enzymes, which can dampen the anti-tumor immune response [23]. Glutamine-depriving conditions compromise the growth and proliferation of activated T cells in vitro [24], whereas restoration of tumor interstitial glutamine levels by tumor-specific GLS1 knockout increases T-cell infiltration and activity in vivo [25]. Indoleamine 2,3-dioxygenase (IDO) is an enzyme that converts tryptophan into kynurenine. Tryptophan depletion and kynurenine accumulation can suppress T cell proliferation and promote the differentiation of Tregs [26]. Arginase 1 (ARG1) is a metabolic enzyme that breaks down the amino acid L-arginine to urea and ornithine; several reports detail how arginine is necessary for the activation and proliferation of CD8^+^ T cells, and some tumor cells can secret arginase to deplete arginine, an amino acid necessary for T cell proliferation and activation [27].

Tumor cells can also alter their lipid metabolism, leading to modulating immune responses. Dysregulation of lipid metabolism is a key characteristic of the TME, and excess lipids in the TME can limit the activity of CD8^+^ T cells, which are important for antitumor immunity [28]. Tumor-derived prostaglandin E2 (PGE2) programs a dysfunctional state in intratumoral type 1 conventional dendritic cells (cDC1s), disabling their ability to locally orchestrate anti-cancer CD8^+^ T cell responses [29]. It also promotes the recruitment and activity of MDSCs [30]. Antigen presentation mainly includes two distinct events: tumor antigens are displayed on the cancer cell surface, and antigen-presenting cells (APCs) take up antigens and cross-present to T cells. Lipids can act as TAAs, directly affecting the immune response during the initial step. Lipids can also modulate antigen presentation through APCs [31].

Many tumor cells express ectonucleotidases like CD39 and CD73, leading to the accumulation of adenosine in the TME. Adenosine can bind to A2A receptors on immune cells, resulting in the inhibition of T cell and NK cell function and the promotion of an immunosuppressive milieu [32].

### Extracellular vesicles (EVs)

EVs derived from cells constitute a diverse set of membrane-bound particles that facilitate communication between cells and their surrounding milieu. These include exosomes, microvesicles, and apoptotic bodies, which are membrane-bound particles carrying a variety of cellular components such as proteins, DNA, lipids, and RNA, and play a role in numerous physiological and pathological processes. In some cases, substances carried by exosomes are similar to cytokines and metabolites, but due to their membrane structure and different mechanisms, they have specific features. Besides protein and metabolites, EVs can also carry a diverse range of molecules, including RNA and DNA.

When exosomes carry programmed death-ligand 1 (PD-L1), they can interact with T cells (specifically the PD-1 receptor on T cells) and inhibit their activation, preventing them from effectively attacking tumor cells [33]. Chen et al. has found that PD-L1 on metastatic melanoma-derived exosomes inhibits the activation of CD8^+^ T cells and facilitates tumor growth, and these effects can be disrupted by anti-PD-1 antibody therapy [34]. PD-L1-positive exosomes inhibit the memory of anti-tumor immune T cells. They induce long-term anti-tumor immune memory and inhibit distant tumor cells through draining lymph nodes, when T cells are transiently exposed to the TME without exosomal PD-L1, and develop a sustained systemic immune response [35]. Exosomes from tumor cells can interfere with the proper presentation of tumor antigens by dendritic cells, hindering their ability to activate T cells effectively [36]. There have been reports showing that exosomes can carry heat shock proteins (HSPs). HSPs released by tumor cells can significantly impact immune cells by suppressing their anti-tumor activity, promoting immune tolerance, and hindering the maturation of immune cells like dendritic cells [37]. Tumor-derived and endogenous exosomal miRNAs can regulate cross-presentation in dendritic cells and with other immune cells; this exosomal miRNAs-mediated intercellular communication may affect the maturation of DCs [38]. NK group 2 member D (NKG2D) is an important activating receptor expressed on the NK cell surface. Acute myeloid leukemia-derived microvesicles can directly decrease NKG2D expression and suppress the activity and cytotoxicity of NK cells [39]. Immature dendritic cells can ingest apoptotic bodies derived from tumor cells, which can lead to immune tolerance, where the immune system becomes less responsive to the tumor antigens, potentially promoting cancer progression [40].

### Extracellular matrix (ECM) proteins

ECM proteins play a crucial role in the TME by influencing cancer progression, metastasis, and immune cell behavior [3]. Luigi et al. demonstrated that lactate secreted by cancer-associated fibroblasts (CAFs) promotes the expression of collagen family genes in prostate cancer. Lactate-utilizing prostate cancer cells rely on elevated α-ketoglutarate (α-KG), which activates α-KG-dependent collagen prolyl-4-hydroxylase to support collagen hydroxylation. This modification enhances stem-like and invasive features by activating discoidin domain receptor 1 (DDR1) [41]. Alterations in the ECM can hinder the tumor infiltration of immune cells, such as T cells, creating an immunosuppressive environment [42, 43]. For instance, increased ECM rigidity has been linked to elevated expression of tenascin-C, which forms a physical barrier that restricts T cell movement and penetration. Additionally, high collagen density in the TME can reduce the cytotoxic activity of tumor-infiltrating immune cells, impair the tumor-killing function of cytotoxic T lymphocytes (CTLs), and facilitate immune evasion by regulating CD8^+^ T cell nuclear size and gene expression [44, 45]. Laminin, a key ECM component, plays a critical role in cell adhesion and signaling, influencing immune cell migration and interaction with tumor tissue. Changes in laminin expression or structure can disrupt these processes, limiting immune cell infiltration and anti-tumor responses [46]. Moreover, a fibrotic ECM directly impacts T cell behavior, often resulting in minimal infiltration in solid tumors and contributing to an immunosuppressive TME [47]. ECM also functions as a reservoir for growth factors and cytokines. Proteins like heparan sulfate proteoglycans bind and modulate the availability of growth factors, influencing cancer cell proliferation and the recruitment of fibroblasts and immune cells [48]. ECM interactions with growth factors regulate their stability and accessibility, playing a pivotal role in cancer progression. This dynamic network undergoes constant remodeling through enzymatic and non-enzymatic post-translational modifications, which alter its structural and functional properties, further shaping its role in the TME [49]. Tumor cells can also remodel the ECM through secreting matrix metalloproteinases (MMPs), potentially facilitating the ingress of immunosuppressive cells like MDSCs and Tregs [50]. Notably, ECM proteins can engage with cell surface receptors on immune cells, activating downstream signaling pathways that may lead to altered immune cell behavior, including changes in migration, differentiation, and cytokine production [51]. Fragments of hyaluronan or fibronectin can activate TLRs, such as TLR2 and TLR4, on macrophages or dendritic cells, leading to pro-inflammatory cytokine production [52].

## Tumor secretome and immune landscape in the TME

**Fig. 2:**
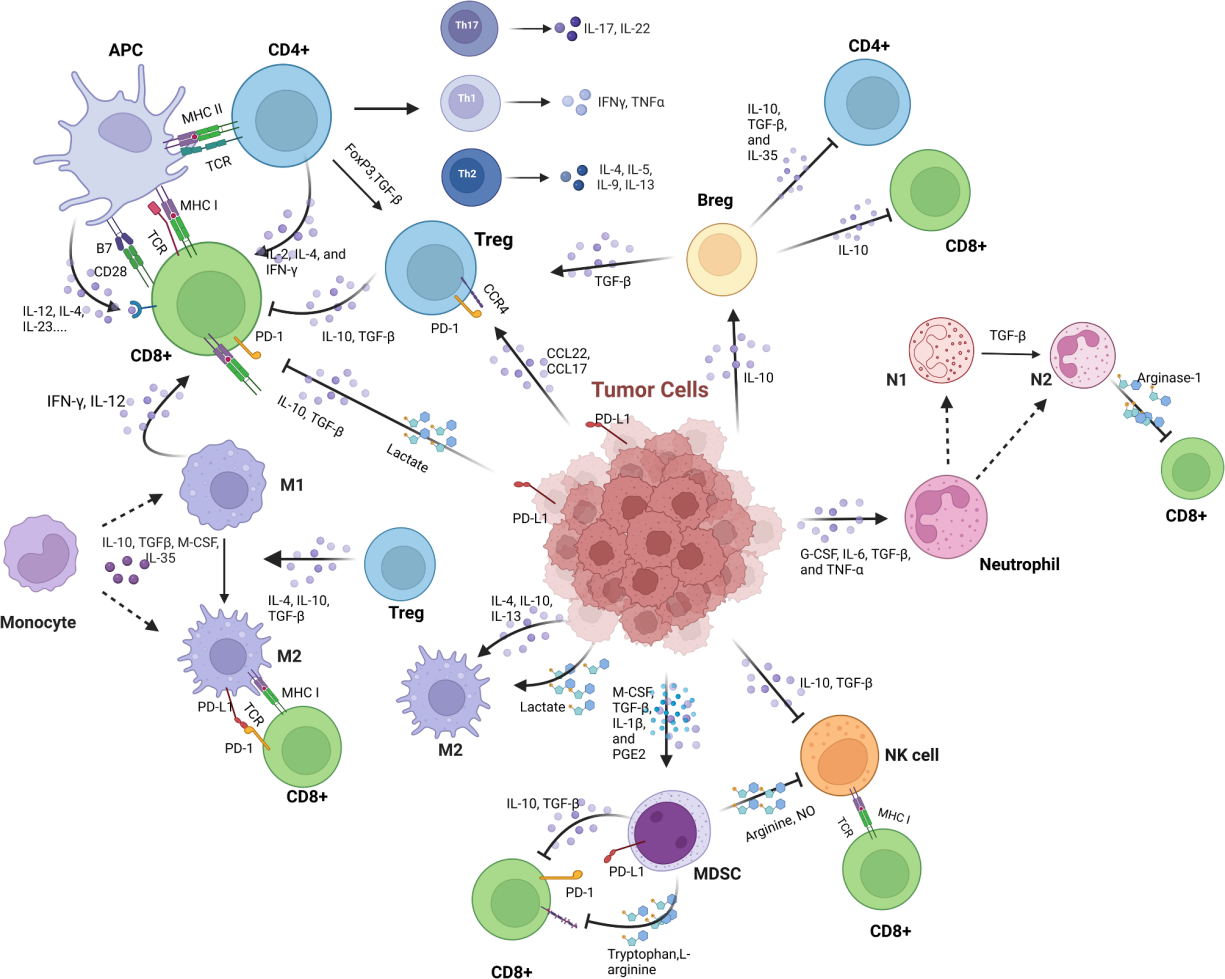
Crosstalk between tumor cells and immune cells. Tumor cells secrete various factors to recruit and activate or inactivate immune cells such as CD8^+^ T cells, CD4^+^ T cells, monocytes, and NK cells. Meanwhile, various immune cells also secrete specific factors to inactivate or inhibit the function of immune cells. This regulation governs the maintenance of the tumor niche, tumor development, and tumor immune function

The immune landscape of a tumor determines its responsiveness to immunotherapy. This landscape refers to the diverse and complex environment of immune cells within and around the tumor. The tumor secretome contributes to this landscape, including the types and abundance of immune cells present, the expression of immune checkpoint molecules, the mutational burden of cancer, and the presence of other immune-modulating factors such as cytokines and chemokines (Fig. 2) [53].

### Tumor secretome and CD8+ T cells

CD8 + T cells, also known as CTLs, are critical for anti-tumor immunity. They exert their tumor-killing function by directly targeting tumor cells and releasing cytotoxic molecules such as perforin, which creates pores in the tumor cell membrane, and granzymes, which enter the cells through these pores to induce apoptosis. The presence of CD8 + T cells within the TME is frequently associated with improved prognosis and enhanced efficacy of immunotherapeutic treatments. Their infiltration into tumors often reflects a robust anti-tumor immune response, contributing to better clinical outcomes [54, 55]. However, tumor cells have evolved various resistance mechanisms to counteract the immune pressure exerted by CD8^+^ T cells, thereby impairing their functionality. These mechanisms include upregulating immune checkpoint molecules, altering antigen presentation pathways, and creating an immunosuppressive TME that inhibits CTL activity. CD8^+^ T cells are essential for recognizing and killing tumor cells via antigen recognition through T-cell receptors (TCRs), In most cancers, elevated PD-L1 expression is typically associated with poor prognosis. Tumor cells secrete PD-L1, which interacts with PD-1 receptors on CD8^+^ T cells, resulting in T-cell exhaustion and diminished cytotoxic activity [56, 57]. Consequently, PD-L1 is regarded as a critical target for immunotherapy strategies in cancer treatment [58]. Tumor cells can also recruit other inhibitory immune cells to suppress immune responses. When CD8^+^ T cells become highly active against a tumor, the tumor can respond by modifying its microenvironment, recruiting immunosuppressive cells such as Tregs and MDSCs. For example, MDSCs can directly interfere with the binding of peptide-MHC (pMHC) complexes to CD8^+^ T cells by nitrating tyrosine residues within the TCR/CD8 complex, preventing CD8^+^ T cells from recognizing pMHC and responding to specific peptides [59]. Additionally, other studies have shown that MDSCs suppress tumor immunity by reducing T cell generation and inducing T cell apoptosis through the activation of apoptotic pathways. This effectively dampens the anti-tumor immune response, enabling the tumor to evade immune destruction. IL-33 derived from tumor-initiating cells recruits macrophages, which, through activator protein 1 (AP-1) signaling, produce TGFβ that suppresses the function of CD8^+^ T cells [60]. Furthermore, surviving tumor cells often downregulate MHC molecules, reducing their visibility to CD8^+^ T cells and limiting immune recognition [61].

### Tumor secretome and CD4+ T cells

CD4^+^ T cells, also known as helper T cells, play a central role in orchestrating the adaptive immune response. Evidence reveals that CD4^+^ T cells play an important role in antitumor immunity by promoting or suppressing CTL responses [62]. Generally, CD4^+^ T cell subsets have varied impacts on tumor growth while they play a pivotal helper role in orchestrating cancer immunity, including Th1, Th2, Th9, Th17, Tregs, CD4^+^ CTL and T follicular helper cells, which present diverse functions, considered to be positive or negative immune cells in antitumor immunity. This indicates that CD4^+^ T cells are more complex than CD8^+^ T cells [63] and have more diverse immune functions. Th1 cells can secrete cytokines like IFN-γ and TNF, which enhance the cytotoxic functions of CD8^+^ T cells and NK cells. Tumor cells can upregulate PD-L1 or secrete IL-10 and TGF-β to counteract the pro-inflammatory and immune-activating effects of Th1 cells [64]. Tregs are crucial for maintaining immune tolerance, preventing autoimmunity, and inhibiting effective anti-tumor immune responses. Tumor cells release chemokines to recruit Tregs and promote their proliferation. Additionally, costimulatory molecules such as CD80/CD86 and CD70 can interact with naïve T cells, facilitating their conversion into Tregs. This process enhances the immunosuppressive environment within the TME, further hindering anti-tumor immune responses [65]. Moreover, Th17 cells, known for their dual roles in immunity, can either promote or inhibit tumor growth depending on the surrounding environment [66]. Tumor cells can suppress Th17 activity through TGF-β or exploit their pro-inflammatory actions to promote tumor-supportive inflammation, angiogenesis, and metastasis. Regulatory B cells (Bregs) are a specialized subset of B cells that contribute to immune tolerance and the prevention of autoimmunity [67]. They promote the differentiation of CD4^+^ T cells into Tregs and secrete immunosuppressive cytokines, including IL-10, TGF-β, and interleukin-35 (IL-35) [68]. IL-35, in particular, has multiple immunosuppressive effects. It not only facilitates Treg differentiation but also disrupts Th1 cell activation, thereby suppressing the activity of differentiated CD8^+^ T cells in anti-tumor responses [69, 70].

### Tumor secretome and NK cells

NK cells are critical for recognizing and eliminating tumor cells through direct cytotoxicity, particularly in cases where tumors lack MHC class I molecules [71]. Tumor cells can reduce the expression of NKG2D ligands, making them less recognizable to NK cells [72]. HLA-E negatively affects the cytotoxic function of both CD8^+^ T cells and NK cells by engaging the inhibitory receptor NKG2A/CD94. Both HLA-G and HLA-E are commonly overexpressed on tumor cells, and expression is inducible by inflammatory cytokines [73]. Certain tumors can actively induce apoptosis in infiltrating NK cells through various mechanisms, such as expressing inhibitory ligands like PD-L1 on their surface and secreting immunosuppressive cytokines [74]. The TME often features hypoxia and nutrient depletion, conditions that impair NK cell metabolism and function. Poznanski et al. found that human NK cell dysfunction in the TME is due to the suppression of glucose metabolism via lipid peroxidation-associated oxidative stress. Activation of the Nrf2 antioxidant pathway restored NK cell metabolism and function and resulted in greater anti-tumor activity in vivo [75].

### Tumor secretome and TAMs

Macrophages are immune cells that can either promote or inhibit tumor growth, depending on their polarization state [76]. Tumor cells secrete chemokines like CCL2, CCL5, and CSF-1 that recruit monocytes from the bloodstream into the TME, where they differentiate into TAMs [77]. TAMs often resemble the M2 macrophage phenotype, which is associated with tissue repair, immune suppression, and tumor promotion. IL-10, TGF-β, and IL-4 secreted from tumor cells drive the polarization of macrophages towards this pro-tumorigenic state [78]. TAMs contribute to immune evasion by suppressing CTL and NK cell functions. They express and secrete immunosuppressive molecules like PD-L1 and arginase, which inhibit T cell activation and proliferation. TAMs further contribute to the suppression of T cell antitumor responses by inducing exhaustion through the upregulation of ligands for exhaustion markers, such as PD-L1 and galectin [79]. Similarly, TAMs can be influenced by metabolic patterns and conditions within the TME. Acidification of the TME, caused by lactate derived from the enhanced glycolytic activity of tumor cells, induces regulatory macrophages through GPCR signaling and IL-1 beta-converting enzyme [80]. This process enhances VEGF and arginase expression, thereby promoting M2-like features of TAMs [81, 82]. IL-35 has been shown to drive macrophages toward an M2-like phenotype, which is associated with anti-inflammatory and tissue-repair roles [83]. These M2-like macrophages contribute to an immunosuppressive TME by secreting anti-inflammatory cytokines such as IL-10 and TGF-β. By promoting a tolerant environment, M2-like macrophages support tumor growth and limit the efficacy of anti-tumor immunity.

### Tumor secretome and MDSCs

MDSCs are a heterogeneous population of immune cells that expand during cancer and other pathological conditions. MDSCs are potent suppressors of the immune response and are often co-opted by tumor cells to promote tumor growth, immune evasion, and relapse after chemotherapy. By fostering an immunosuppressive environment, MDSCs inhibit the activity of anti-tumor immune cells, including CD8^+^ T cells, and contribute to resistance to therapeutic interventions, making them a significant target for cancer treatment strategies [4, 10, 84]. Therefore, targeting MDSCs represents an attractive therapeutic strategy to enhance cancer immunotherapy. MDSCs expand and migrate to the TME through the interaction between CCR and CXCR and their corresponding chemokine ligands. CXCR2 secreted from tumor cells plays a crucial role in the migration of MDSCs into the TME and the pre-metastatic niche. Targeting CXCR2 can reduce the recruitment of MDSCs to the TME thereby diminishing their immunosuppressive influence [85, 86]. Moreover, tumor cells secrete various cytokines and chemokines, such as granulocyte-macrophage colony-stimulating factor (GM-CSF), granulocyte colony-stimulating factor (G-CSF), macrophage colony-stimulating factor (M-CSF), IL-6, IL-10, and PGE2, to promote the expansion and recruitment of MDSCs from the bone marrow to the tumor site [87, 88]. Targeting the signaling pathways responsible for the differentiation and expansion of MDSCs, such as those mediated by tumor-secreted factors like GM-CSF, IL-6, and VEGF, can block MDSC development and maturation [89, 90]. Tumor cells also stimulate MDSCs to produce thrombospondin 1 (THBS1), which contributes to the development of a metastasis-resistant microenvironment. Mechanistically, prosaposin secreted by tumor cells activates pro-metastatic, bone marrow-derived MDSCs to secrete THBS1, fostering the creation of a metastasis-refractory environment in distant organs [91]. This highlights prosaposin and THBS1 as potential therapeutic targets for inhibiting tumor metastasis. Additionally, tumor cells secrete metabolites such as lactate, adenosine, and kynurenine, which directly influence the development, function, and expansion of MDSCs [92]. Targeting these metabolic pathways, particularly those involving lactate or adenosine production, can help alleviate the suppressive conditions created by MDSCs and improve anti-tumor immunity [93].

## Research approaches and clinical applications for the study of the tumor secretome

**Fig. 3:**
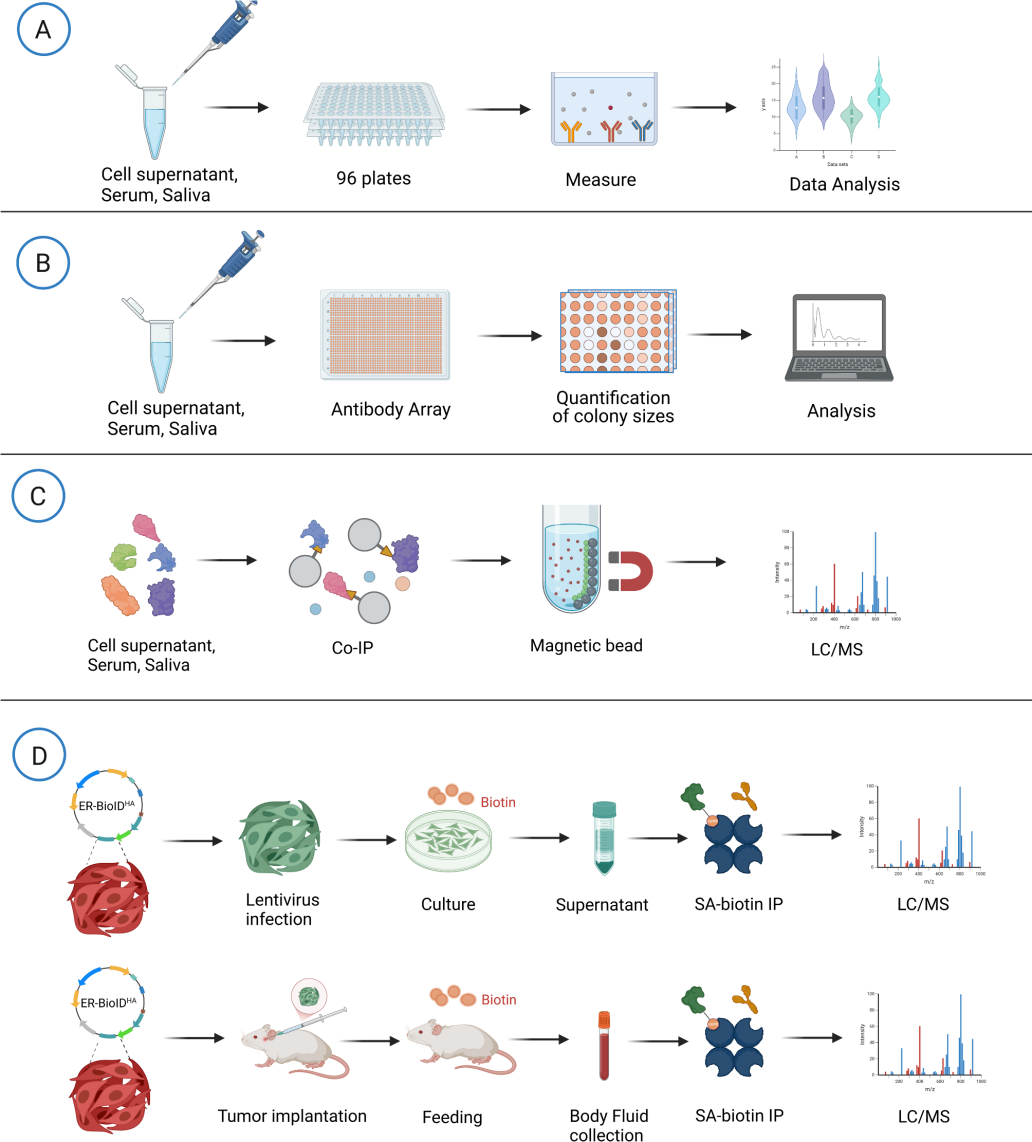
Overview of the main methods for detecting tumor secretome in research. (**A**) ELISA. Fluids are collected and placed on ELISA plates that have been pre-coated with a specific capture antibody. The target protein adheres to this antibody, and then an enzyme-linked detection antibody is introduced. The protein’s concentration is determined by measuring the colorimetric shift that occurs when the enzyme reacts with its substrate. (**B**) Antibody arrays. Samples containing secreted proteins from cell culture or body fluids are incubated with precoated antibody arrays. Specific antibodies immobilized on the array capture various secreted proteins. Bound proteins are detected using a reporter molecule conjugated to a secondary antibody, allowing simultaneous quantification of multiple proteins in a single sample and providing a comprehensive secretome profile. (**C**) Mass spectrometry after immunoprecipitation. After collection and concentration of cell culture supernatants or body fluids, secretome immunoprecipitation is performed. Biomolecules of interest are isolated and analyzed by mass spectrometry to identify and quantify secreted proteins and metabolites. (**D**) ER-BioID^HA^ biotinylation technique. The ER-BioID^HA^ plasmid is transfected into tumor cells to enable biotinylation of secreted proteins. Biotin is added to the culture and secreted proteins are biotinylated in situ. Streptavidin-based immunoprecipitation is performed to isolate the biotinylated proteins, which are then analyzed by mass spectrometry to identify a specific set of secreted molecules

The study of the tumor secretome offers valuable insights into tumor progression and the identification of potential biomarkers. Various detection approaches are available to analyze the tumor secretome, each providing unique advantages in characterizing the molecular profile and dynamics of secreted proteins and other biomolecules (Fig. 3). Techniques such as two-dimensional gel electrophoresis and mass spectrometry are frequently used to analyze proteins in the secretome, facilitating the identification of promising cancer biomarkers. For instance, Brandi et al. discussed how proteomics can uncover cancer cell-specific secretions, providing deeper insights into cancer cell behavior and their interactions with the TME [94]. Another way to study the secretome is by culturing tumor cells in vitro. Isolated proteins from cancer cell supernatant can be analyzed to identify the tumor secretome components [95]. Researchers aim to correlate secretome components with blood biomarkers by combining secretome analysis with serum biomarker identification. Xue et al. demonstrated this approach to explore serum-based biomarkers that reflect tumor progression [96]. Another study explored the use of proteomic techniques to compare secretomes across different cancer stages, providing insights into cancer progression and identifying secreted molecules [97]. These approaches to tumor secretome research allow for a more comprehensive understanding of the complex interactions within the TME and aid in developing therapeutic interventions targeting these pathways.

Identifying the cellular or tissue origin of proteins derived from serum remains a significant challenge. To capture the tumor-specific secretome while minimizing the influence of serum proteins, an ER lumen-resident BioID expression plasmid (ER-BioID^HA^) has been developed [98]. ER-BioID^HA^ expression plasmid was designed to contain the BioID coding sequence with the following features in order: IgK signal peptide, BioID2 coding sequence, HA tag, and the ER retention signal KDEL (Lys-Asp-Glu-Leu) tetrapeptide. Using proximity biotinylation, this advanced system tracks tumor cell-secreted proteins or circulating proteins, enabling precise identification of tumor-specific secreted molecules. This approach provides a more accurate understanding of the molecular factors involved in tumor biology and the TME. Our lab recently leveraged this system to identify specific secreted proteins from tumor cells infected with wild-type vesicular stomatitis virus (wtVSV) or VSV expressing human *SMAC/DIABLO* gene (VSV-S) [99]. The results from this analysis demonstrate that VSV-S has a greater potential to induce pyroptosis in head and neck cancer cells than wtVSV.

Understanding the tumor cell secretome is crucial for advancing cancer research, as it enables the development of targeted therapies, enhances early detection methods, and allows for the design of personalized treatment strategies based on specific molecular targets. Table 1 presents a summary of studies investigating the tumor secretome in clinical trials.

**Table 1:** Clinical trials exploring tumor secretome-based therapies in solid tumors

NCT Identifier	Target	Phase	Cancer Type	Treatment	Status
NCT01575340	IL-10	Not Applicable	Colorectal cancer	Encapsulated fish oil	Completed
NCT02009449	IL-10	Phase I	Prostate cancer, ovarian cancer, breast cancer	Pegilodecakin, oxaliplatin, pazopanib, nivolumab, gemcitabine	Completed
NCT05991583	IL-10	Phase I	Advanced and metastatic solid tumors	IBB0979	Recruiting
NCT06468358	IL-10	Phase II	Advanced and metastatic solid tumors	LB1410, LB4330	Recruiting
NCT06044311	TGF-β	Phase II	Esophageal Adenocarcinoma	Vactosertib	Not yet recruiting
NCT04481256	TGF-β	Not Applicable	Esophageal cancer	Paclitaxel, carboplatin	Recruiting
NCT01112293	TGF-β	Phase II	Melanoma	GC1008	Completed
NCT02947165	TGF-β	Phase I	Lung cancer, breast cancer, hepatocellular carcinoma, colorectal cancer, pancreatic cancer, renal cell carcinoma	NIS793, PDR001	Completed
NCT02937272	TGF-β	Phase I	Advanced and metastatic solid tumors	LY3200882, LY3300054, gemcitabine, nab-Paclitaxel, cisplatin	Active
NCT04432597	TGF-β	Phase II	Oropharyngeal cancer	M7824	Active
NCT02581787	TGF-β	Phase II	Lung cancer	Fresolimumab	Completed
NCT02065362	TGF-β	Phase I	Nasopharyngeal carcinoma	TGF-β resistant cytotoxic T-lymphocytes	Active
NCT06026657	TGF-β	Phase II	Breast cancer	Gemcitabine, TGF-βi NK Cells	Recruiting
NCT04574583	TGF-β	Phase II	Breast cancer, head and neck squamous cell cancer	M7824, SX-682, CV301 TRICOM	Completed
NCT05588648	TGF-β	Phase II	Osteosarcoma	Vactosertib	Recruiting
NCT04247282	TGF-β	Phase II	Head and neck squamous cell carcinoma	M7824	Completed
NCT05637216	TGF-β	Phase II	Breast cancer	Losartan	Recruiting
NCT03206177	TGF-β	Phase I	carcinosarcoma of the uterus and ovary	Galunisertib, paclitaxel, carboplatin	Completed
NCT01401062	TGF-β	Phase II	Breast cancer	Fresolimumab	Completed
NCT06579196	TGF-β2	Phase II	Lung cancer	Trabedersen, pembrolizumab	Not yet recruiting
NCT02178358	TGF-β	Phase II	Hepatocellular Carcinoma	LY2157299, sorafenib	Completed
NCT03620201	TGF-β	Phase I	Breast cancer	M7824	Completed
NCT05400122	TGF-β	Phase I	Colorectal cancer	Vactosertib, fludarabine phosphate, cyclophosphamide, IL-2, NK cells	Recruiting
NCT02734160	TGF-β	Phase I	Pancreatic cancer	Galunisertib, durvalumab	Completed
NCT06203912	TGF-β	Phase I	Myeloma	TGFbi NK cells, isatuximab	Recruiting
NCT02160106	TGF-β	Phase I	Melanoma, breast cancer, hepatocellular carcinoma, prostate cancer	TEW 7197	Completed
NCT04551950	TGF-β	Phase I	Cervical cancer	M7824, carboplatin, paclitaxel, bevacizumab, cisplatin	Completed
NCT02688712	TGF-β	Phase II	Rectal cancer	LY2157299, capecitabine, fluorouracil	Active
NCT00356460	TGF-β	Phase I	Renal cancer, malignant melanoma	GC1008	Completed
NCT02423343	TGF-β	Phase II	Solid tumor, lung cancer, hepatocellular carcinoma	Galunisertib, nivolumab	Completed
NCT03833661	TGF-β	Phase II	Cholangiocarcinoma, gallbladder Cancer	M7824	Completed
NCT06637306	IL-4	Phase I	Breast cancer	Dupilumab, pembrolizumab, paclitaxel, carboplatin	Not yet recruiting
NCT05967884	IL-4	Phase II	Breast cancer	Cemiplimab, dupilumab	Not yet recruiting
NCT00433446	IL-6	Phase II	Prostate cancer	CNTO 328	Completed
NCT00841191	IL-6	Phase II	Sarcoma	CNTO 328	Completed
NCT05704634	IL-6	Phase I	Lung cancer	Sarilumab, cemiplimab	Recruiting
NCT05233397	IL-6	Phase II	Adamantinomatous Craniopharyngioma	Tocilizumab	Recruiting
NCT01637532	IL-6	Phase II	Ovarian cancer	Carbo, caelyx, carbo,​doxorubicin, tocilizumab	Completed
NCT00402181	IL-6	Phase II	Myeloma	CNTO 328	Completed
NCT04729959	IL-6	Phase II	Glioblastoma	Tocilizumab, atezolizumab, radiation	Active
NCT05428007	IL-6	Phase II	Myeloma	Sarilumab, ipilimumab, nivolumab, relatlimab	Recruiting
NCT03999749	IL-6	Phase II	Myeloma	Tocilizumab, ipilimumab, nivolumab	Active
NCT01484275	IL-6	Phase II	Myeloma	Siltuximab	Completed
NCT04940299	IL-6	Phase II	Melanoma, lung cancer, urothelial carcinoma	Tocilizumab, ipilimumab, nivolumab	Active
NCT05391750	IL-6	Phase I	Myeloma	Venetoclax, tocilizumab	Recruiting

## Challenges and perspectives

Research into the tumor secretome holds great potential for advancing innovative cancer treatments. However, effectively leveraging this approach presents both substantial challenges and significant opportunities. Studies highlight the high heterogeneity of the tumor secretome, with different cancer types and subtypes exhibiting diverse secretome profiles [100]. This variability complicates the development of standardized therapeutic approaches and the identification of reliable biomarkers, necessitating personalized profiling techniques for effective application. Furthermore, the secretome is dynamic, changing in response to environmental conditions, treatment pressures, and cancer progression. Identifying key therapeutic targets within this variability is complex, and ensuring their relevance over time as the secretome evolves introduces an additional challenge. Moreover, not all secreted factors are equally crucial; targeting less important molecules may result in limited efficacy or unintended side effects. Critically, the safety of targeting the secretome demands thorough evaluation, as these therapies may affect not only tumor cells but also normal physiological processes due to the diverse roles of secretome components.

To overcome these challenges, comprehending and capitalizing on the tumor secretome can give rise to more precise and efficacious therapeutic strategies. Several crucial viewpoints need to be emphasized to enhance the effects of tumor treatment. Firstly, aside from the biomarkers mentioned earlier, specific proteins or metabolites secreted by tumor cells should be investigated in accordance with different tumors, even for individual patients. Based on various secretome profiles, clinicians can customize therapies to target these distinct features, resulting in more effective and individualized treatment options. Secondly, monitoring strategies need to be established to detect the tumor secretome, which might circulate in bodily fluids that mirror tumor dynamics and treatment responses. Thirdly, given the existence of treatment resistance, uncovering new targeting molecules involved in tumor progression and metastasis to serve as potential therapeutic targets is of paramount significance. Comprehending the role of these molecules in promoting cancer growth or facilitating immune evasion can furnish information for the development of novel drugs that inhibit these pathways.

**Fig. 4:**
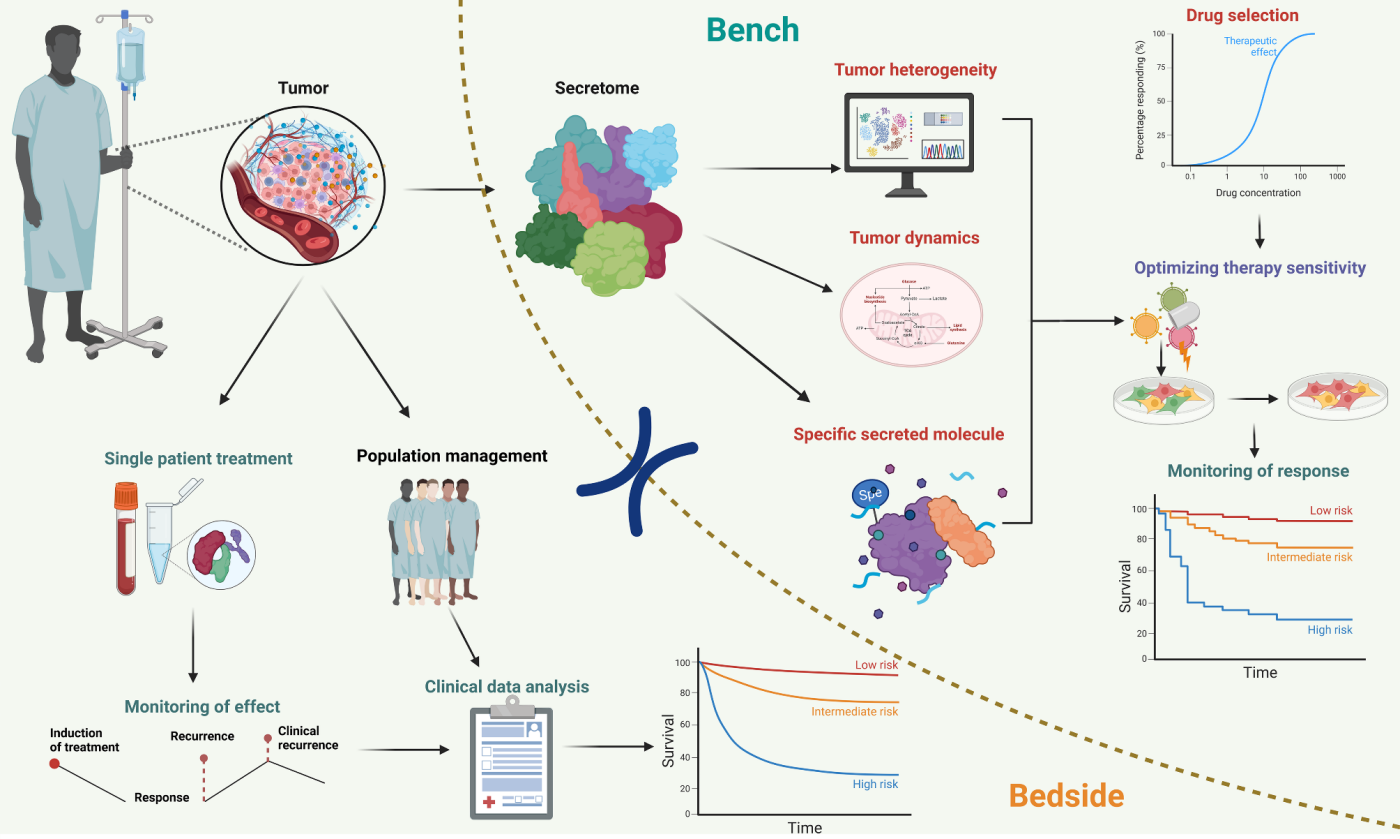
Overview of the bench-to-bedside and bedside-to-bench approaches for personalized treatment and patient management through tumor secretome analysis. The secretome profiles of individual patient tumors are analyzed to identify unique molecular signatures. In the laboratory, these tumors are subjected to thorough screening for potential drug candidates, taking into account their inherent heterogeneity, dynamic metabolic profiles, and specific protein expression patterns. Drug sensitivity assays are performed to optimize therapeutic efficacy and assess patient response. Clinically, treatment recommendations are based on laboratory findings, allowing for personalized therapy adjustments. Treatment efficacy is carefully monitored and the risk of relapse is assessed. Large patient cohorts are monitored and extensive clinical follow-up is performed, using advanced data analytics to refine treatment strategies and improve outcomes

The ultimate goal of basic research in the field of cancer is to guide the development of innovative clinical treatment strategies. In this context, we also propose a personalized treatment process focused on targeting the tumor secretome. This involves obtaining tumor samples from cancer patients and analyzing changes in the secretome to address the challenges posed by tumor heterogeneity and distinct metabolic dynamics. We aim to identify specific targeting molecules for tumor treatment. Through comprehensive drug screening, we can optimize drug efficacy by selecting compounds that specifically target these molecules. By monitoring the effects of these drugs in real time, we can refine personalized drug regimens in clinical practice, ensuring that they align with the unique metabolic profile of each patient’s tumor. This method not only guides personalized drug use but also contributes to the development of individualized treatment plans. By implementing such tailored strategies, we hope to improve patient management and treatment outcomes, achieving comprehensive improvements in the clinical care of cancer patients (Fig. 4).

## Conclusion

In conclusion, the tumor secretome plays a crucial role in regulating the TME. This review highlighted how the tumor secretome influences the immune landscape within tumors, demonstrating its potential to either promote immune evasion or enhance anti-tumor immunity. Understanding these regulatory effects offers new opportunities for therapeutic interventions, with a focus on reprogramming the tumor secretome to boost immune responses and inhibit tumor progression.

## Data Availability

No datasets were generated or analysed during the current study.

## Abbreviations

TME: Tumor microenvironment
TGF-β: Transforming growth factor beta
EMT: Epithelial-mesenchymal transition
IL-10: Interleukin-10
IL-35: Interleukin-35
NK: Natural killer cells
CXCR1: C-X-C motif chemokine receptor 1
MDSCs: Myeloid-derived suppressor cells
TAMs: Tumor-associated macrophages
MHC: Major histocompatibility complex
Treg: Regulatory T cells
Breg: Regulatory B cells
IDO: Indoleamine 2,3-dioxygenase
ARG1: Arginase 1
PGE2: Prostaglandin E2
cDC1s: Type 1 conventional dendritic cells
APCs: Antigen-presenting cells
EVs: Extracellular vesicles
PD-L1: Programmed Death-Ligand 1
aPD-1: Anti-PD-1
HSPs: Heat shock proteins
NKG2D: NK group 2 member D
ECM: Extracellular matrix
CTLs: Cytotoxic T lymphocytes
MMPs: Matrix metalloproteinases
TCRs: T-cell receptors
AP-1: Activator protein 1
GPCR: G protein-coupled receptor
GM-CSF: Granulocyte-macrophage colony-stimulating factor
G-CSF: Granulocyte colony-stimulating factor
M-CSF: Macrophage colony-stimulating facto
MCTs: Monocarboxylate transporters
α-KG: α-ketoglutarate
VSV: Vesicular stomatitis virus
THBS1: Thrombospondin 1
